# The impact of mindfulness meditation on social and moral behavior: Does mindfulness enhance other-oriented motivation or decrease monetary reward salience?

**DOI:** 10.3389/fnint.2022.963422

**Published:** 2022-09-02

**Authors:** Susanna Feruglio, Maria Serena Panasiti, Cristiano Crescentini, Salvatore Maria Aglioti, Giorgia Ponsi

**Affiliations:** ^1^Italian Institute of Technology, Sapienza University of Rome and CLN^2^S@Sapienza, Rome, Italy; ^2^Department of Languages, Literatures, Communication, Education, and Society, University of Udine, Udine, Italy; ^3^Department of Psychology, Sapienza University of Rome, Rome, Italy; ^4^IRCCS Santa Lucia Foundation, Rome, Italy; ^5^Institute of Mechanical Intelligence, Scuola Superiore Sant’Anna, Pisa, Italy

**Keywords:** mindfulness meditation, social behavior, moral behavior, compassion, other-oriented motivation, reward salience, self-control, resistance to temptation

## Abstract

This perspective article provides an overview of the impact of mindfulness meditation (MM) on social and moral behavior. In mindfulness research, prosocial behavior has been operationalized as helping behavior, altruistic redistribution of funds, reparative behavior, or monetary donation. Studies concerning moral behavior are still scarce. Despite inconsistent evidence, several studies found a beneficial effect of mindfulness on prosocial outcomes (i.e., a higher propensity to spend or give away money for the sake of other individuals). However, since the employed tasks were reward-based, participants’ decisions also directly affected their own payoff by reducing it. Crucially, MM also affects self-control circuitry and reduces reward-seeking behaviors and reward salience by making rewards less tempting. We have discussed evidence suggesting how challenging it may be to dissociate the specific weight of enhanced other-oriented motivation from one of the decreased monetary reward salience in explaining meditators’ behavior. Future higher-quality studies are needed to address this open issue.

## Introduction

The scientific interest in mindfulness meditation (MM) has significantly increased in the last two decades due to its well-documented beneficial effects on physical (Greeson and Chin, 2019) and psychological health (Brown et al., 2015). MM is a practice that relies upon techniques of mental training, encouraging individuals to focus, with an open, gentle, and self-compassionate attitude, on their internal experiences, such as bodily sensations, thoughts, and emotions (Lutz et al., 2008). The mechanisms of action through which MM exerts its effects are multiple: attentional control, emotion regulation, body awareness, and change in perspective on the self (Hölzel et al., 2011).

Neuroimaging evidence shows that MM might induce neuroplastic changes in the structure and the function of brain regions involved in attentional processes, memory, emotion regulation, self-awareness, exteroceptive and interoceptive body awareness, and self-control (Fox et al., 2014; Tang et al., 2015; but see Kral et al., 2022 for a different view). Other meditation practices include loving-kindness meditation (LKM, aimed at developing positive emotions toward oneself and others) and compassion meditation (CM, aimed at cultivating compassion toward others) (Hofmann et al., 2011). A summary of the most employed terms is reported in Table 1. Crucially, MM seems to positively affect compassion (Condon et al., 2013; Lim et al., 2015), and also CM-based interventions have been found to enhance mindfulness (Jazaieri et al., 2014; Brito-Pons et al., 2018).

**TABLE 1:** Glossary: A summary table with the description of the acronyms and the definitions of the most important terms used in the perspective article.

Term	Acronym	Definition
Mindfulness meditation	MM	Meditation practice based upon techniques of mental training encouraging individuals to focus, with an open, gentle and non-judgmental attitude, on their internal experiences such as bodily sensations, thoughts, and emotions (Lutz et al., 2008).
Compassion meditation	CM	Meditation practice aimed at enabling practitioners to volitionally generate states of compassion in response to their own and others’ suffering while maintaining a positive emotional state (Engen and Singer, 2014).
Loving-kindness meditation	LKM	Meditation practice aimed at self-generating positive emotions, feelings of love, warmth and goodwill toward oneself and others (Kok et al., 2013).
Dispositional mindfulness	DM	Also called trait mindfulness. It refers to the stable tendency to pay attention to present moment experience with an open and nonjudgmental attitude (Brown and Ryan, 2003).
Prosocial behavior	−	Any action that benefits one or more people other than the actor (Pfattheicher et al., 2022), characterized by distinct underlying motivations: other-oriented altruistic motivation, self-oriented egoistic motivation, norm-based motivation, and/or strategic motivation (Böckler et al., 2016).
Moral behavior	−	Any course of action performed in line with norms, values, and customs adopted by a specific cultural group (e.g., consideration of actions’ direct or indirect consequences for others; Moll et al., 2005; Ayala, 2010).

Although MM has classically investigated the individual domain, recent investigations have tried to understand whether the beneficial effects of mindfulness practice can extend individual boundaries, reaching the domain of social and moral traits and behaviors.

## The impact of mindfulness on socio-moral stance

Morality refers to a system of norms, values, and customs adopted by a specific cultural group to guide socio-moral behavior (Moll et al., 2005). Moral behavior refers to the ability to perform a course of action in line with moral norms, e.g., by considering its direct or indirect consequences for others (Ayala, 2010). Morality can be assessed distinctly by measuring moral judgments (e.g., by means of moral dilemmas, such as the Trolley and the Footbridge dilemmas (Nichols and Mallon, 2006; Christensen and Gomila, 2012), actual moral behavior (e.g., by means of interactive tasks requiring participants to actively take decisions or perform actions in morally relevant contexts, with real consequences for oneself and others, e.g., the Temptation to Lie Card Game; Panasiti et al., 2011, 2014, 2016; Azevedo et al., 2017; Schepisi et al., 2020; Scattolin et al., 2022; Vabba et al., 2022), or trait morality (e.g., the Moral Identity Questionnaire; Black and Reynolds, 2016).

Recently, it was proposed that mindful states may impact the ability to detect morally relevant information and, consequently, moral behavior (Sevinc and Lazar, 2019). Nonetheless, only a few studies probed the effects of mindfulness on morality. Georgiou et al. (2020) investigated the longitudinal association among dispositional mindfulness (DM, a stable tendency to pay attention to present moment experience with an open and non-judgmental attitude), impulsivity, moral disengagement, and bullying. The authors found that DM had an indirect effect on both bullying and victimization, which is fully mediated by reduced impulsivity and moral disengagement. Xiao et al. (2020) recently explored the relationship between DM, moral judgment, and prosocial behavior. In a cross-sectional study (*N* = 554), the authors found positive correlations among DM, moral sensitivity, moral identity, and prosocial tendencies. In a randomized controlled experiment (*N* = 99), they investigated the effect of an 11-week mindfulness intervention on moral identity and prosocial behavior in participants who underwent the training (*N* = 49) and in the wait-list control group (*N* = 50). The results showed that mindfulness practice improved self-compassion, also affecting prosocial behavior in high moral identity participants (Xiao et al., 2020). Small and Lew (2021) employed a survey to measure DM, moral responsibility, and moral judgment (*N* = 171). They found that DM predicted moral responsibility but not moral judgment. These studies suffer from methodological limitations, such as the employment of self-reports or hypothetical scenarios instead of the measurement of actual behavior in a morally relevant context.

Prosocial behavior can be defined as any action that benefits one or more people other than the actor (Pfattheicher et al., 2022) and is characterized by distinct underlying motivations: other-oriented altruistic motivation (e.g., an action that benefits another individual but is neutral or costly for the actor), self-oriented egoistic motivation (e.g., strategic action that benefits another individual but is rewarding also for the actor), norm-based motivation (e.g., an action performed to comply to or enforce social norms), and/or strategic motivation (e.g., action based on cost-benefit calculations) (Böckler et al., 2016; Schindler and Friese, 2022).

Prosociality can be assessed by means of different measures (Böckler et al., 2016), such as game theoretical paradigms (e.g., economic games, such as the Dictator Game), interactive computer tasks (e.g., donation tasks), hypothetical distribution tasks (e.g., social discounting), or psychological trait questionnaires (e.g., Prosocialness scale; Caprara et al., 2005).

Meta-analytical evidence on the link between mindfulness and prosocial behavior reported mixed findings (Kreplin et al., 2018; Luberto et al., 2018; Donald et al., 2019; Berry et al., 2020). Luberto et al. (2018) showed that MM-based interventions increased empathy, compassion, and prosocial behavior. In fact, meditation training induced, with respect to control groups, a greater increase in at least a prosocial (subjective or objective) outcome measure in 22 out of 26 studies. The authors also highlighted that the effects were stronger and more consistent for observable outcomes (e.g., actual helping behavior) than self-reported ones (Luberto et al., 2018). Similarly, Donald et al. (2019) found a positive relationship between mindfulness (as both a trait and an intervention) and prosociality. The authors found no evidence for a greater effect on helping behavior of interventions explicitly focused on the cultivation of prosocial emotions with respect to those focused on mindful awareness (Donald et al., 2019). This last pattern was corroborated by another meta-analysis, which highlighted that MM without explicit ethics-based instructions promoted compassionate (but not instrumental or generous) helping behavior (Berry et al., 2020). Despite these positive findings, Kreplin et al. (2018) showed that the effects of MM on prosocial behavior were limited, also highlighting the weak methodological quality of most of the reviewed studies (61%). The authors found a moderate increase in compassion and empathic behavior following MM, but no effects on the other considered behaviors (aggression, connectedness, and prejudice) (Kreplin et al., 2018). Furthermore, other studies found evidence of a negative impact of mindfulness on prosociality (Chen and Jordan, 2020; Guo et al., 2021; Hafenbrack et al., 2021; Poulin et al., 2021). Crucially, the inconsistency of the meta-analytical evidence on MM and prosocial behavior might be due to methodological issues and biases (e.g., employment of self-report instead of behavioral measures and use of correlational instead of longitudinal studies) (Schindler and Pfattheicher, 2021; Schindler and Friese, 2022).

## Mindfulness and socio-moral behavior as measured with reward-based tasks

In mindfulness research, prosocial behavior has often been operationalized as helping behavior (Leiberg et al., 2011; Condon et al., 2013; Lim et al., 2015), altruistic redistribution of funds (Weng et al., 2013, 2015), financial allocation (Hafenbrack et al., 2020), reparative behavior (Hafenbrack et al., 2021), or monetary donation (Ashar et al., 2016; Chen and Jordan, 2020; Iwamoto et al., 2020; Schindler and Pfattheicher, 2021).

Here, we highlighted a potential problem in the interpretation of the effects of mindfulness on socio-moral behavior, namely, the employment of behavioral tasks that involve some kind of incentive or reward for the participants (see Ponsi et al., 2021 for a discussion about the link between reward motivation and moral behavior). In fact, in both games, theoretical paradigms and interactive computer tasks, prosocial decisions directly affect participants’ actual payoff (Böckler et al., 2016). A summary of the tasks employed in the reviewed studies is reported in Table 2.

**TABLE 2:** Description and categorization of the tasks employed in the studies reported in the perspective article.

Studies	Task	Task type	Description	Measured behavior	Prosocial behavior motivation	Category
Leiberg et al. (2011) and Böckler et al. (2018)	Zurich prosocial game	Interactive computer task	The participant decides whether to help or not a co-player to open gates during a virtual maze navigation task aimed at reaching distinct treasures worth 0.50 Swiss francs in a limited amount of time (absence of competition between the players).	Helping behavior	Altruistically-motivated	Reward-based (money)
Weng et al. (2013)	Redistribution game	Game theoretical paradigm	After witnessing an unfair dictator transfer ($1/$10) to a cashless victim, the participant decides whether to spend any amount of their endowment ($5) to compel the dictator to give two times the amount to the victim. The participant is paid the amount left in their endowment.	Helping behavior, altruism	Norm-motivated	Reward-based (money)
Weng et al. (2015) and Böckler et al. (2018)	Third-party punishment game	Game theoretical paradigm	After witnessing an unfair dictator transfer (< $2.50/$10) to the Recipient, the participant (third party) decides whether to spend any amount out of $5 (50 points) to take two times the amount from the dictator. The participant is paid the amount left in their endowment.	Punishment behavior	Norm-motivated	Reward-based (money)
Weng et al. (2015)	Third-party helping game	Game theoretical paradigm	After witnessing an unfair dictator transfer (< $2.50/$10) to the recipient, the participant (third party) decides whether to spend any amount out of $5 (50 points) to transfer two times the amount to the recipient. The participant is paid the amount left in their endowment.	Helping behavior, altruism	Norm-motivated	Reward-based (money)
Hafenbrack et al. (2020, Study 2b)	Financial allocation task	Interactive computer task	The participant decides whether to allocate any amount of a hypothetical lottery win between themselves and another participant. The participant knows that the donated amount would be multiplied by 1.5. The one participant who wins the lottery receives a real €120 payoff.	Altruism, generosity	Altruistically-motivated	Reward-based (money)
Böckler et al. (2018); Chen and Jordan (2020), Iwamoto et al. (2020), and Schindler and Pfattheicher (2021, Study 1)	Donation task	Interactive computer task	The participant decides whether to donate any (or the entire) amount of their monetary endowment to a charitable organization. The participant is paid the amount left in their endowment.	Donation, charitable behavior	Altruistically-motivated	Reward-based (money)
Ashar et al. (2016, Study 2)	Charitable donation task	Interactive computer task	The participant decides whether to donate a portion of their own experimental earnings to each of the 24 individuals in need, from $0 to $100 in $1 increments. One of their donations was randomly selected and subtracted from their endowment.	Donation, charitable behavior	Altruistically-motivated	Reward-based (money)
Böckler et al. (2018) and Schindler and Pfattheicher (2021, Study 2)	Dictator game	Game theoretical paradigm	The participant decides whether to give a fraction of their financial outcome to another player.	Altruism, generosity	Altruistically-motivated	Reward-based (money)
Böckler et al. (2018)	Trust game	Game theoretical paradigm	The participant decides whether to invest an amount of money to the Trustee that is multiplied by some factor (often 3). The trustee then chooses an amount to send back to the participant which decides the payoff for both players.	Trust, Strategic behavior	Altruistically-motivated	Reward-based (money)
Böckler et al. (2018)	Second party punishment game	Game theoretical paradigm	After playing the role of dictator, the participant plays the role of recipient and decides whether to spend any amount of monetary units (1 MU = 10 eurocent) to remove three times the amount from the dictator. The participant is paid the amount left in their endowment.	Punishment behavior	Norm-motivated	Reward-based (money)
Condon et al. (2013) and Lim et al. (2015)	Ecologically valid staged scenario	−	The participant is exposed to a real-life situation in which a suffering confederate with crutches and a walking boot entered a waiting area without available chairs. If the participant offers their seat in the next 2 min, their behavior is coded as helping; otherwise, it is coded as non-helping.	Helping behavior, compassionate responding	−	Non-reward-based
Berry et al. (2018)	Cyberball game (inclusion)	−	After witnessing a ball tossing game in which a player was ostracized from two other players, the participant played with them. The proportion of the total throws that the participant makes to the victim is coded as inclusion behavior.	Inclusion behavior	−	Non-reward-based
Berry et al. (2018)	E-mail helping	−	After witnessing a ball-tossing game (Cyberball game) in which a player was ostracized from two other players, the participant writes an e-mail to them. Responses to the victim, coded for communication warmth, served as a measure of helping and support behaviors.	Helping behavior	−	Non-reward-based

Task type and prosocial behavior motivation columns include the labels proposed by Böckler et al. (2016, 2018). Accordingly, task type and prosocial behavior motivation content only apply to reward-based tasks.

Previous evidence highlighted that CM practice may directly affect prosocial behavior (but see Condon, 2019 for a review of moderating variables). Leiberg et al. (2011) developed a task called Zurich Prosocial Game (ZPG) aimed at assessing helping behavior toward strangers. Participants’ task was to navigate a virtual character through a maze and reach a treasure in a limited amount of time. Each treasure was worth 0.50 Swiss francs. In the same maze, there was another co-player trying to reach a different treasure, so the players were not competing. Crucially, during the maze navigation, participants had the opportunity to help the co-players to open their gates. Participants were informed that their goal was to optimize their monetary gains. The results of Experiment 2 showed that short-term CM increased helping behavior in the ZPG, compared with short-term memory training, and that helping in the no-reciprocity trials was correlated to the reported practice hours in the CM group (Leiberg et al., 2011). In addition, Böckler et al. (2018) tested the effect of different trainings and found that LKM boosted altruistically motivated prosocial behavior but not norm-motivated behavior.

A similar behavioral pattern emerged in studies investigating altruistic redistribution of money. Weng et al. (2013) employed the redistribution game to investigate whether a 2-week CM training could affect altruism. During the game, participants witnessed an unfair economic interaction (i.e., a dictator, endowed with $10 who transferred $1 to a victim who had no money). Participants could decide to spend any amount of their endowment ($5) to compel the dictator to give two times the amount to the victim. Participants were paid the amount left in their endowment after this decision. The results showed that CM training increased altruistic behavior with respect to reappraisal training. In addition, participants were presented with images of human suffering and non-suffering during functional MRI (fMRI) scans before and after the trainings. In the compassion group, greater inferior parietal cortex activation during the processing of human suffering was associated with increased redistribution (Weng et al., 2013). Similarly, Weng et al. (2015, Study 2) investigated whether a 2-week CM training could impact two different facets of altruistic behavior, punishment, and helping. In the first interaction, the dictator (endowed with 100 points) could choose to transfer any amount to the recipient (endowed with 0 points). In the second interaction of the Punishment Game, the participant (endowed with 50 points) could decide to spend points to deduct points from the dictator. In the Helping Game, the participant (endowed with 50 points) could decide to spend points to transfer points to the recipient. Importantly, game points were converted to dollars (10 points = $1), and each player was paid accordingly. The authors found that a 2-week online CM training increased altruistic helping of victims (but not altruistic punishment of wrongdoers) compared to an active reappraisal training (Weng et al., 2015).

Other studies investigated the impact of mindfulness on donation and charitable behavior. Recently, Iwamoto et al. (2020) used a donation task in which participants were informed about the option to transfer part of their experimental endowment to a charity organization. Participants who underwent an MM online session donated 2.61 times more money with respect to the ones who underwent the control online session (Iwamoto et al., 2020). Similarly, Hafenbrack et al. (2020) employed a financial allocation task in which participants could decide to donate part of a hypothetical win in a real lottery (€120) to another participant (Study 2b). Individuals who were engaged in a focused breathing MM task were more generous with respect to mind-wandering control participants (Hafenbrack et al., 2020). In addition, Chen and Jordan (2020) asked participants whether they would donate any of their experimental payoff ($15 Canadian) to a charity organization. The authors compared the effects of a 6-day meditation practice with (EthicalM) or without (SecularM) additional ethical instructions. The results indicated that EthicalM, compared to SecularM, increased the amount of money donated to a charity and that this effect was moderated by trait empathy for both protocols (Chen and Jordan, 2020). Other studies found no effects of MM and DM on donation behavior. For example, Ashar et al. (2016, Study 2) employed a charitable donation task to assess the effects of a smartphone-based CM program on prosocial outcomes. Participants were given the possibility to donate a portion of their own experimental payoff ($100) to each of the 24 suffering individuals. Participants who followed the CM training did not increase charitable donations with respect to control interventions (Ashar et al., 2016). Similarly, Schindler and Pfattheicher (2021) found no significant relationship between DM and prosocial behavior by measuring donation behavior to a charitable organization (Study 1) and giving behavior in the Dictator Game (Study 2).

To summarize, some of the above-reviewed studies showed a beneficial effect of MM, CM, and LKM trainings on several prosocial outcomes (despite the studies investigating donation behavior reporting the most inconsistent findings). Since the employed tasks were reward based, this translates into a higher propensity to spend or give away money for the sake of other individuals after various types of training.

## Mindfulness, self-control, and reward salience

As briefly mentioned in the introduction, MM also impacts an individual’s cognitive functioning (see, for example, Feruglio et al., 2021; Lin et al., 2021). Repeated mental training activities are known to benefit the efficiency of attentional processing (van den Hurk et al., 2010; Becerra et al., 2017), attention-related behavioral responses (Jha et al., 2007), and to affect neurophysiological measures of executive attention, such as the event-related potential P3 (Lin et al., 2019). Further, MM seems to increase the efficiency of cognitive control and conflict monitoring (Larson et al., 2013; Jo et al., 2017), to improve self-regulation (Tang et al., 2007; Friese and Hofmann, 2016; Kaunhoven and Dorjee, 2017), emotion regulation (Teper et al., 2013; Roemer et al., 2015; Tang et al., 2016), self-control (Bowlin and Baer, 2012; Friese et al., 2012), and to reduce impulsivity (Hendrickson and Rasmussen, 2013, 2017; Yao et al., 2017; Dixon et al., 2019).

Mindfulness-based interventions also seem to influence basic reward processing. Kirk and Montague (2015) employed a passive conditioning task during fMRI in a group of experienced mindfulness meditators and age-matched controls to investigate whether the practice of MM influences reward and reward prediction error (PE) signals. They found diminished positive and negative PE-related brain responses in the putamen (part of the reward network) of meditators when compared with controls (Kirk and Montague, 2015).

Crucially, mindful attention seems to modulate the relationship between motivation and behavior. Participants trained to observe their moment-by-moment reward-driven mental states reported reduced neural and self-reported craving for smoking images (Westbrook et al., 2013) and decreased effects of motivational states/traits on appetitive behavior in sex and food reward domains (Papies et al., 2015).

The beneficial effects of MM interventions on reward salience are also supported by studies in addiction neuroscience. Garland et al. (2014) found that mindfulness-oriented recovery enhancement (MORE) induced a decrease in opioid use and craving during the treatment, decreased subjective opioid cue-reactivity after the treatment, and impacted cardiac-autonomic responsiveness toward a reward. Recently, Garland et al. (2019) showed that MORE affected reward processing (Froeliger et al., 2017) and decreased participants’ opioid cue-reactivity as indexed by reduced late positive potential (LPP, an event-related potential modulated by attention to emotional information).

Pivotal for reward-driven behavior, mindfulness-based interventions help to reduce different kinds of behavioral addictions (Brewer et al., 2013), such as smoking (Tang et al., 2013), maladaptive eating behaviors (Mason et al., 2015, 2016; Shomaker et al., 2019), and drug addiction (Bowen et al., 2014; Garland, 2021).

## Discussion

The reviewed studies investigating the effect of mindfulness on prosocial behavior share a common feature: the employment of reward-based behavioral tasks that directly affect participants’ payoff. The presented evidence often shows a beneficial effect of mindfulness and compassion on several prosocial outcomes, which may reflect a higher propensity to spend or give away money for the sake of other individuals.

Since mindfulness also contributes to improved self-control and reduced reward salience, we argue that the employment of reward-based tasks may make it difficult to properly dissociate the specific contribution to prosociality of enhanced other-oriented motivation (operationalized as a higher amount of money given in favor of others) from decreased individual reward salience (operationalized again as a higher amount of money given in favor of others). In other words, does mindfulness induce people to give money to others for their sake or make money less tempting, making it easier to give it away?

It is worth noting that not all studies investigating the effect of mindfulness on prosocial behavior adopted reward-based tasks. Condon et al. (2013) exposed participants enrolled in an 8-week MM training to a real-life situation in which a person with crutches was suffering. They found that meditators offered their seat more often than non-meditators. Similarly, Lim et al. (2015) used the same real-life situation and showed that participants who took part in a 3-week mobile-app MM training gave up their seats more frequently than controls who were enrolled in a cognitive skills course. Further, Berry et al. (2018) found that both DM and a brief mindfulness-based training increased inclusion and helping behavior toward ostracized strangers. These studies suggest that mindfulness practice may target specifically other-oriented motivation even in the absence of manifest rewards for the participants. Crucially, behaving prosocially consistently activates the reward circuit (Cutler and Campbell-Meiklejohn, 2019) and positively impacts individuals’ reputations (Berman and Silver, 2022). Then, the employment of non-reward-based tasks does not exclude a partial contribution of self-oriented motivation in the development of these behaviors.

Prosocial behavior relies on several mental processes, such as socio-cognitive and socio-affective ones (Preckel et al., 2018). A recent study suggests that the brain networks through which distinct meditation practices may affect prosocial behavior comprise the prefrontal and anterior cingulate cortices (cognitive control and conflict processing) in breathing and body scan meditation; the inferior frontal and lateral temporal cortices (cognitive perspective-taking) in observing-thoughts meditation; and the frontal and insular regions (empathy and emotion regulation) in LKM (Valk et al., 2017).

To prevent interpretation biases, future studies should opt for experimental designs that parallelly manipulate both other-oriented motivation and monetary reward salience. They should also investigate the dynamic development of both processes, since they may both positively affect the socio-moral stance but with different timings or trajectories. Finally, future research should investigate whether the prosocial effects of distinct mindfulness training activities may derive from different psychological mechanisms: an increase in other-oriented motivation for LKM/CM and an enhancement in self-control for MM practice.

## Data availability statement

The original contributions presented in this study are included in the article/supplementary material, further inquiries can be directed to the corresponding author.

## Author contributions

All authors listed have made a substantial, direct, and intellectual contribution to the work, and approved it for publication.
